# Third-trimester uterine rupture following previous salpingectomy for interstitial pregnancy: radiologic and surgical insights

**DOI:** 10.1186/s12884-025-07919-z

**Published:** 2026-02-10

**Authors:** Po-Fan Chen, Yu-Ling Liang

**Affiliations:** 1 Department of Obstetrics and Gynecology, College of Medicine, National Cheng Kung University Hospital, National Cheng Kung University, No. 138, Sheng Li Road, Tainan, Taiwan; 2 Graduate Institute of Clinical Medicine, College of Medicine, National Cheng Kung University Hospital, National Cheng Kung University, Tainan, Taiwan

**Keywords:** Uterine rupture, Interstitial pregnancy, Salpingectomy, Third trimester, MRI, Ultrasonography, Uterine surgery, Interpregnancy interval

## Abstract

**Background:**

Uterine rupture in the third trimester is a rare but life-threatening obstetric emergency, with greater likelihood in pregnant patients with a history of uterine surgery.

**Case presentation:**

We present a unique case of uterine rupture at 30 weeks gestation in a patient with a recent surgical history of laparoscopic salpingectomy involving myometrial resection for interstitial ectopic pregnancy, who conceived five months after the procedure. This case provides valuable radiologic and surgical insights, highlighting the importance of imaging modalities in diagnosis and the potential risks associated with short interpregnancy intervals following prior uterine surgery.

**Conclusions:**

Early detection and prompt intervention are crucial for favorable maternal and fetal outcomes.

## Background

Uterine rupture (UR) is a catastrophic obstetric event that poses significant risks to both mother and fetus. It is commonly associated with prior uterine surgery that results in myometrial scarring and structural weakening of the uterine wall [1–3]. Interstitial pregnancies, a rare form of ectopic pregnancy occurring in the proximal part of the fallopian tube that penetrates the muscular layer of the uterus, require surgical intervention that may weaken the uterine wall and lead to scar tissue formation [4–6]. Such scar tissue typically lacks the tensile strength and elasticity of normal myometrium, potentially compromising the structural integrity of the uterus. The optimal interpregnancy interval after such surgeries is not well established, but shorter intervals may increase the risk of UR due to insufficient healing time and incomplete remodeling of the uterine wall and scar tissue [7–9].

We report a case of uterine rupture at 30 weeks gestation in a patient with a recent uterine surgical history, who conceived five months after surgery. This case emphasizes the critical role of imaging in diagnosis and raises awareness about the potential risks associated with early conception following uterine surgery.

## Case presentation

**Fig. 1:**
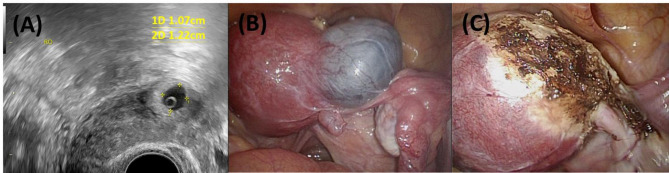
Imaging and surgical views of previous right interstitial pregnancy. **A** Transvaginal ultrasound image showing a right interstitial ectopic pregnancy. **B** Laparoscopic view of the right interstitial pregnancy before salpingectomy. **C** Laparoscopic view after salpingectomy, showing the surgical site post-resection

A 36-year-old woman, gravida 2 para 0, presented to our emergency department at 30 weeks and 2 days of gestation with acute right lower quadrant abdominal pain. Five months prior to conception, she had undergone a laparoscopic salpingectomy with resection of the right interstitial portion for an interstitial ectopic pregnancy (Fig. 1). The surgery involved partial myometrial resection, and her postoperative course was uneventful.

At presentation, her vital signs were stable, and fetal heart rate monitoring was reassuring. She denied any trauma, vaginal bleeding, or contractions. Her current pregnancy had progressed without complications until the sudden onset of abdominal pain. She was initially evaluated by the emergency physician, with acute appendicitis suspected. Approximately 30 min later, a transabdominal ultrasound was performed, which revealed an abnormal protrusion on the right lateral aspect of the uterus, raising concern for possible uterine wall defect and fetal part herniation. She was then referred to the obstetric team for further evaluation, and an obstetric ultrasound was immediately performed, supporting the suspicion of uterine rupture. Within approximately 50 min, magnetic resonance imaging (MRI) was performed, confirming a uterine defect. Emergency surgery was arranged approximately 30 min after imaging confirmation.

### Imaging findings

**Fig. 2:**
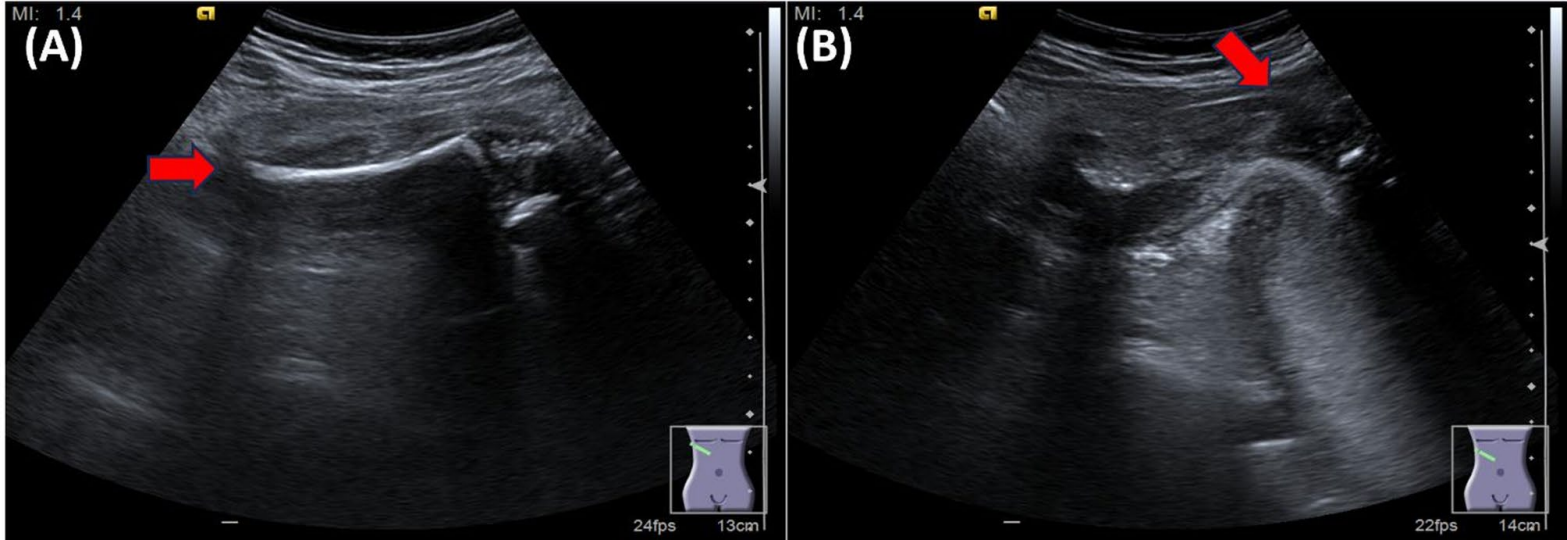
Ultrasonographic findings suggestive of uterine rupture. **A** Transabdominal ultrasound image of the maternal right upper abdomen showing a suspected fetal thigh protruding outside the uterine contour (red arrow). **B** Transabdominal ultrasound image of the right uterine myometrium showing suspected fetal thigh protrusion through a uterine defect (red arrow indicates the area of myometrial discontinuity)

**Fig. 3:**
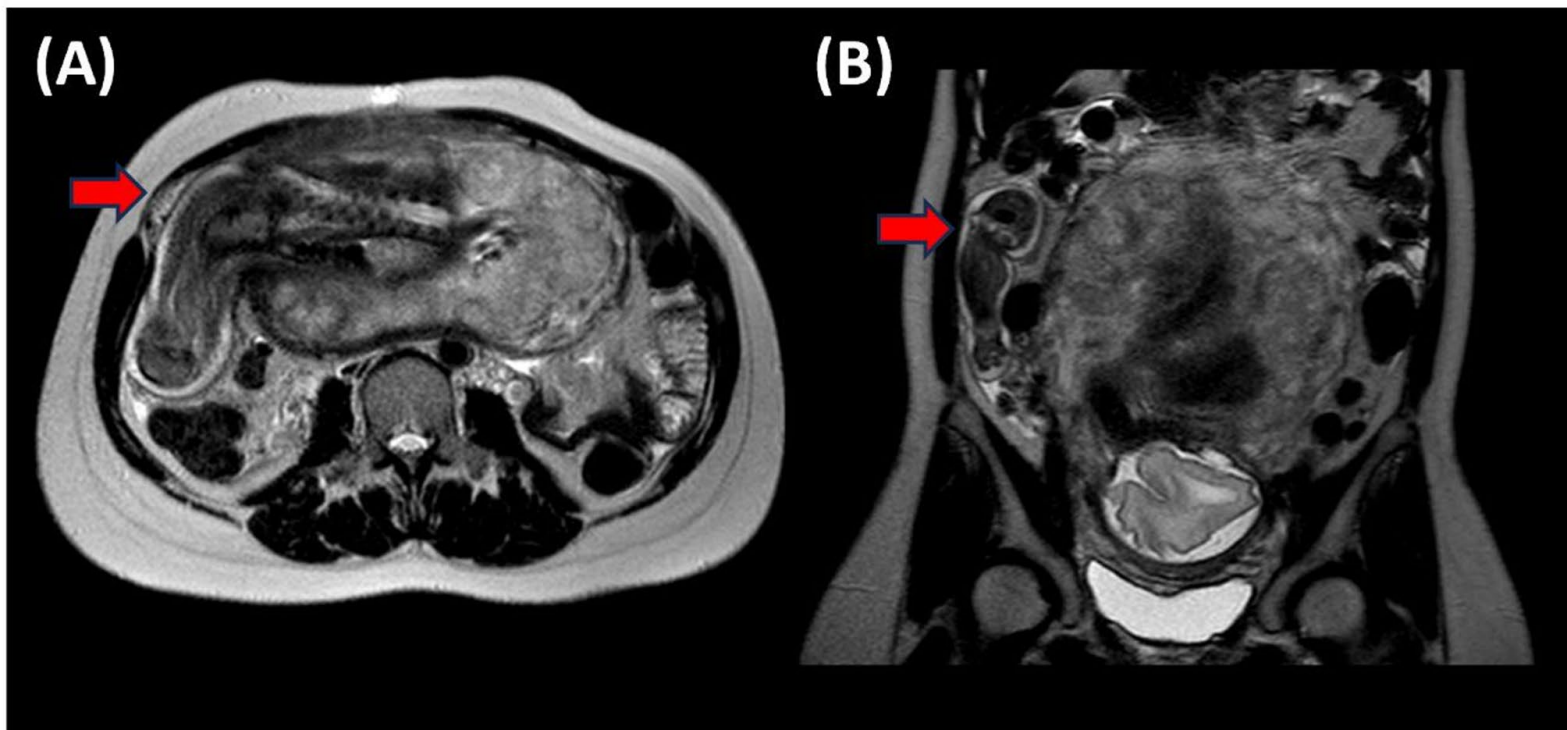
MRI confirmation of uterine rupture. **A** Axial MRI image demonstrating uterine rupture at the right interstitial region with fetal parts herniating through the uterine wall (arrow indicates the site of rupture). **B** Sagittal MRI image showing the uterine rupture and protrusion of fetal parts (arrow indicates the area of uterine wall defect and herniation)

**Fig. 4:**
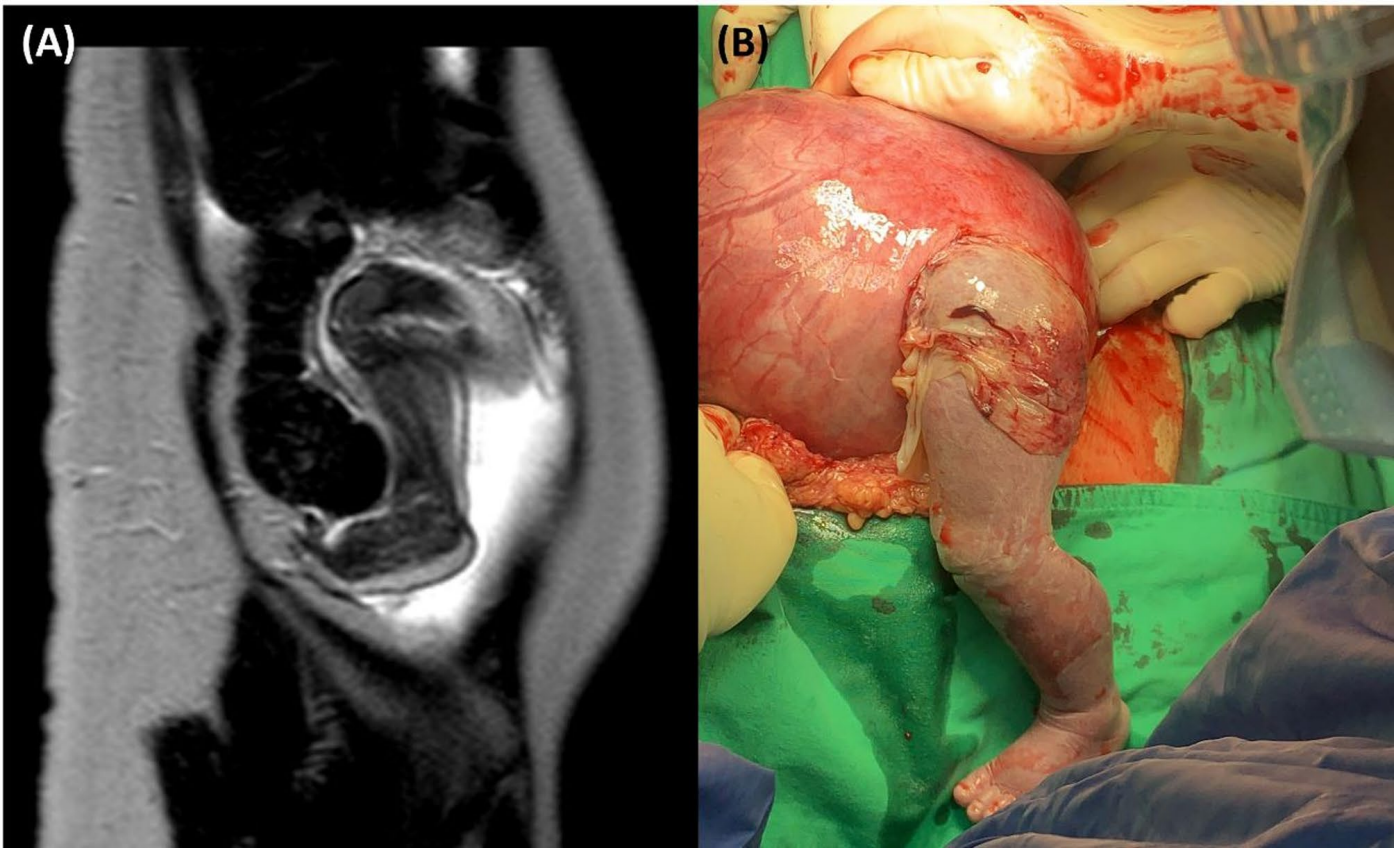
MRI and intraoperative images of uterine rupture with protruding fetal leg. **A** MRI image showing uterine rupture at the right interstitial region with the fetal leg protruding through the uterine wall (red arrow). **B** Intraoperative view confirming uterine rupture at the previous surgery site with the fetal leg protruding through the uterine defect (white arrow)

Initial transabdominal ultrasonography revealed an abnormal protrusion on the right lateral aspect of the uterus (Fig. 2). Detailed ultrasonography suggested herniation of fetal parts through a uterine defect. MRI was performed for further evaluation, confirming a uterine rupture at the right interstitial region with the fetal leg protruding through the uterine wall (Figs. 3 and 4A). The protruding fetal part appeared to lack membranous covering, suggestive of a ruptured amniotic membrane. Intraoperative findings demonstrated a ruptured amniotic membrane, with the fetal leg protruding freely through the uterine defect (Fig. 4B).

### Surgical intervention

**Fig. 5:**
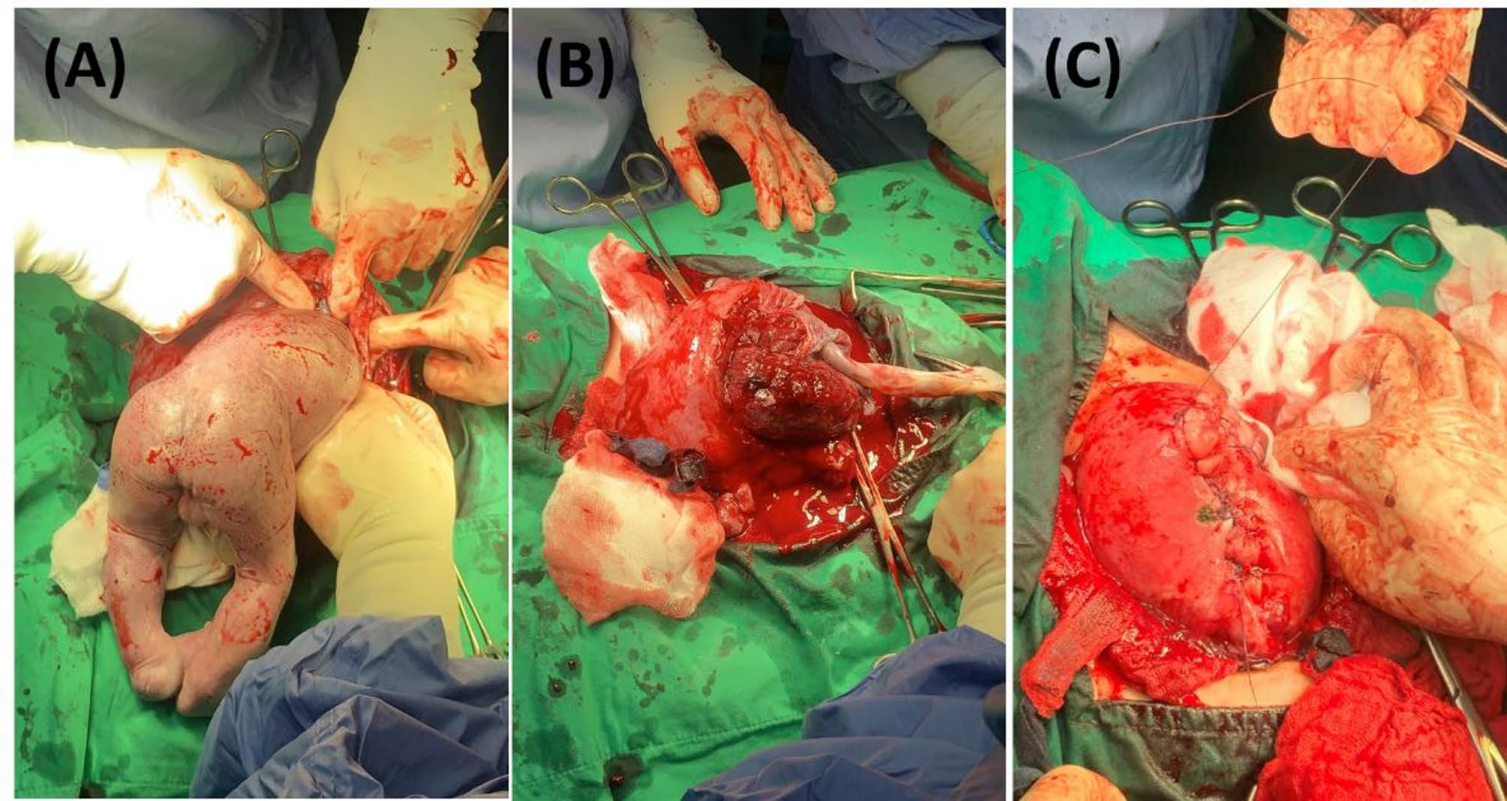
Delivery and repair process through the uterine rupture site. **A** Delivery of the fetus through the uterine rupture site. **B** Delivery of the placenta through the same rupture site. **C** Repair of the uterine myometrium in two layers using absorbable sutures

An emergency laparotomy was undertaken due to the diagnosis of uterine rupture. Intraoperative findings confirmed a 5 cm rupture at the site of prior uterine surgery with fetal leg herniation (Fig. 4B). The amniotic membrane was noted to be ruptured intraoperatively, with the fetal limb directly exposed to the maternal peritoneal cavity. A classical cesarean section was performed through the rupture site, delivering a male infant weighing 1,500 g with Apgar scores of 8 and 9 at 1 and 5 min, respectively (Fig. 5A and B).

The uterine rupture site was repaired in two layers using absorbable sutures (Fig. 5C). The estimated blood loss was approximately 800 mL, and the patient received two units of packed red blood cells intraoperatively. The mother’s postoperative course was uneventful, and she was discharged on postoperative day 8. The neonate was admitted to the neonatal intensive care unit and discharged in stable condition on postnatal day 38.

## Discussion

This case highlights a rare occurrence of uterine rupture in the third trimester following a prior uterine surgery. The rupture occurred at the site of the prior uterine surgery, suggesting a possible link between the sufficient length of time for myometrial healing and the mechanical stress of the growing pregnancy.

### Risk factors and healing time

The risk of UR is known to increase in patients with uterine surgical history due to scar formation and decreased myometrial integrity [1–3]. The healing of the uterine incision or resection site is crucial for maintaining uterine strength in subsequent pregnancies. Although there are no definitive guidelines for the recommended interpregnancy interval after prior uterine surgery, some literature suggests that waiting at least 6–12 months may reduce the risk of UR by allowing adequate myometrial healing [7–9].

### Literature evidence

Tong et al. reported a case of asymptomatic uterine rupture in the second trimester following laparoscopic surgery for interstitial pregnancy, emphasizing the importance of ultrasound in early detection [10]. Similarly, Feng et al. [11] described a spontaneous complete uterine rupture with protrusion of fetal limbs in the third trimester after laparoscopic cornuostomy, highlighting the potential risk of rupture associated with prior uterine surgeries. Additionally, Ahlschlager et al. discussed the challenges in diagnosing late-presenting ruptured interstitial pregnancies, underscoring the need for heightened clinical vigilance [12]. These cases collectively emphasize the potential risks associated with short interpregnancy intervals and the importance of appropriate imaging modalities in diagnosis.

### Clinical consideration and recommendations

Based on the present case and supporting literature, it may be reasonable to consider advising patients to delay conception for at least 6–12 months following uterine surgeries involving myometrial resection [7–9]. While definitive guidelines are lacking, a longer interpregnancy interval may allow more complete myometrial healing and potentially reduce the risk of UR in subsequent pregnancies. Further research with larger case series or cohort studies is needed to confirm this association. In the meantime, physicians may consider performing individualized risk assessments and thorough evaluations of uterine integrity before future pregnancies.

### Imaging modalities

The use of MRI was pivotal in diagnosing the uterine rupture preoperatively. While ultrasonography is the first-line imaging modality in obstetrics, MRI offers superior soft-tissue contrast and a larger field of view, which can be critical in complex cases. Aboughalia et al. highlighted the value of imaging in evaluating uterine perforation and rupture, suggesting that MRI can provide detailed information that influences management decisions [13]. However, MRI should only be utilized when the maternal and fetal conditions are stable to avoid delays in urgent care.

### Clinical implications

This case raises awareness of the potential risks associated with early conception following prior uterine surgery involving myometrial resection. Clinicians may consider discussing interpregnancy timing with patients in similar clinical contexts, as shorter intervals from surgery to conception may increase risks. While further research is needed to determine definitive counseling guidelines, clinicians may consider discussing interpregnancy timing with patients in similar clinical contexts, as early conception may increase risks. Additionally, patients with a uterine surgical history should be monitored closely during subsequent pregnancies, especially in the third trimester. As highlighted in the cases reported by Ahlschlager et al. [12] and Tong et al. [10], delayed diagnosis of uterine rupture can lead to severe maternal and fetal complications.

### Suggested clinical actions

- Interpregnancy Timing: Based on this case and related reports [8, 9], clinicians may consider discussing with patients who have a history of uterine surgery the potential risks. While high-quality evidence is lacking, it is suggested as an expert opinion that a 6–12 month interpregnancy interval following myometrial resection may allow more complete uterine healing and potentially reduce the risk of rupture.
- Preconception Evaluation: For patients with a history of uterine surgery, it may be recommended to assess uterine integrity and other related risk factors before subsequent pregnancies.
- Pregnancy Surveillance: As supported by this case and other reports of uterine rupture following myometrial surgery [10–12], closer clinical and sonographic monitoring during pregnancy in patients with prior uterine surgery involving myometrial resection may help in earlier detection of complications.
- Imaging Use: MRI may be helpful in selected cases when ultrasonography findings are inconclusive and when it does not delay urgent care, especially when UR is suspected [13].

## Conclusions

Uterine rupture in the third trimester following prior uterine surgery involving myometrial resection is an obstetric rarity with significant implications. This case illustrates the critical role of imaging in diagnosis and management and raises concern that conception within five months after uterine surgeries involving myometrial resection may be associated with an increased risk of rupture. Adequate healing time and careful monitoring are essential to prevent such life-threatening complications.

## Data Availability

The data presented in this study are available from the corresponding author upon reasonable request.
